# TLR and NKG2D Signaling Pathways Mediate CS-Induced Pulmonary Pathologies

**DOI:** 10.1371/journal.pone.0078735

**Published:** 2013-10-09

**Authors:** Brian W. Wortham, Bryan L. Eppert, Jennifer L. Flury, Sara Morgado Garcia, Michael T. Borchers

**Affiliations:** Division of Pulmonary, Critical Care, and Sleep Medicine, Department of Internal Medicine, University of Cincinnati College of Medicine, Cincinnati, Ohio, United States of America; French National Centre for Scientific Research, France

## Abstract

Long-term exposure to cigarette smoke (CS) can have deleterious effects on lung epithelial cells including cell death and the initiation of inflammatory responses. CS-induced cell injury can elaborate cell surface signals and cellular byproducts that stimulate immune system surveillance. Our previous work has shown that the expression of ligands for the cytotoxic lymphocyte activating receptor NKG2D is enhanced in patients with COPD and that the induction of these ligands in a mouse model can replicate COPD pathologies. Here, we extend these findings to demonstrate a role for the NKG2D receptor in CS-induced pathophysiology and provide evidence linking nucleic acid-sensing endosomal toll-like receptor (TLR) signaling to COPD pathology through NKG2D activation. Specifically, we show that mice deficient in NKG2D exhibit attenuated pulmonary inflammation and airspace enlargement in a model of CS-induced emphysema. Additionally, we show that CS exposure induces the release of free nucleic acids in the bronchoalveolar lavage and that direct exposure of mouse lung epithelial cells to cigarette smoke extract similarly induces functional nucleic acids as assessed by TLR3, 7, and 9 reporter cell lines. We demonstrate that exposure of mouse lung epithelial cells to TLR ligands stimulates the surface expression of RAET1, a ligand for NKG2D, and that mice deficient in TLR3/7/9 receptor signaling do not exhibit CS-induced NK cell hyperresponsiveness and airspace enlargement. The findings indicate that CS-induced airway injury stimulates TLR signaling by endogenous nucleic acids leading to elevated NKG2D ligand expression. Activation of these pathways plays a major role in the altered NK cell function, pulmonary inflammation and remodeling related to long-term CS exposure.

## Introduction

Long-term exposure to cigarette smoke (CS) leads to a progressive decline in pulmonary function and can ultimately result in the onset of diseases such as chronic obstructive pulmonary disease (COPD). COPD is a complex disease characterized by alterations in airway epithelial cells, peribronchial and perivascular inflammation and permanent alveolar enlargement [1]. Cellular subsets of both the innate and adaptive immune response coordinate inflammation and tissue destruction contributing to the pathogenesis of COPD [2]. Understanding the specific mechanisms by which these lymphocyte subpopulations contribute to the altered balance between epithelial cell injury and repair is an important focus of COPD research.

Our previous work demonstrated a novel role for natural killer (NK) cells in the development of COPD [3-5]. NK cells are considered sentinel cells of the immune system because of their ability to target stressed and infected cells without prior activation. The interaction of NK cells with target cells involves an orchestrated engagement of activating and inhibiting receptors. Of the activating receptors, NKG2D (*Klrk1*) is distinguished as it is present on virtually all NK cells, targets stress- and infection-induced ligands, and can overcome inhibitory signals to mediate cytotoxicity and cytokine release [6]. NKG2D recognizes a diverse array of ligands on the cell surface including MICA, MICB, and the UL-16 binding proteins in humans and the retinoic acid–inducible early transcripts (Raet1α–Raet1ε), H60, and Mult1 in mice [6]. NKG2D ligand expression is absent on cell surfaces under normal conditions. However, ligands are induced under conditions of stress, DNA damage, infection and transformation. In the case of NK cells, engagement of NKG2D causes lysis of ligand expressing cells and cytokine production, most notably IFN-γ [7]. In the context of CS exposure, NKG2D ligands are induced on stressed human airway epithelial cells [8], and persistently expressed on pulmonary epithelial cells of smokers with and without COPD [3]. RAET1 expression is similarly induced in the alveolar and airway epithelium in a mouse model of COPD [3].

Utilizing data obtained from COPD patients and a mouse model of lung specific NKG2D ligand expression, we have shown that the activation of the NKG2D receptor is a potentially important contributor to lung inflammation and tissue destruction in COPD [3]. Specifically, conditional expression of NKG2D ligands in pulmonary epithelial cells in a transgenic mouse was sufficient for the development of emphysema [3] and mice deficient in NKG2D fail to develop enhanced pulmonary remodeling following influenza infection in a model of COPD [5]. Although these findings suggest multiple NK cell functions are attributable to NKG2D in tissue remodeling following CS exposure and during viral exacerbations, the necessity of NKG2D and the mechanisms that contribute to the activation of NKG2D-bearing cells remains to be elucidated.

An important pathway involved in the activation of NK cells is mediated through a subset of cell surface/endosomal Toll-like receptors (TLR) [9] and cytoplasmic pattern recognition receptors [10] found on accessory cells. TLRs are activated during viral infection based on the recognition of viral pathogen associated molecular patterns (PAMPs). Stimulation of TLR3, 7, or 9 expressed by dendritic cells (DCs) and other accessory cells leads to NK cell activation through production of type I IFNs, IL-12, IL-18, and IL-15 [9,11]. Activation of NK cells is important to control viral infections due to their ability to directly kill virus-infected cells [12]and noncytotoxic functions such as cytokine production [13]. More recently, accumulating evidence details TLR activation to other molecules that are not pathogen derived. During tissue injury, necrotic cell death causes the loss of membrane integrity and the release of intracellular contents. These cellular contents, which include various species of nucleic acids [unmethylated CpG DNA (TLR9), and ssRNA/mRNA (TLR3/7)], can stimulate TLR receptors [14]. This class of molecules is collectively termed Damage Associated Molecular Patterns (DAMPs). There is evidence that signaling through these TLRs initiates and amplifies pathogen-independent responses. For example, TLR3 has been implicated in lung inflammation and injury associated with hyperoxia [15].

In this study, we examined the relationship of endogenous ligands released from CS exposure and the roles of NKG2D and nucleic acid-sensing TLRs in the development of COPD pathologies. We show that mice deficient in NKG2D or mice deficient in TLR3/7/9 receptor signaling have reduced inflammation and pulmonary remodeling when compared to their filtered air (FA)-exposed controls. We show that mice deficient in TLR3/7/9 signaling exhibit attenuated NKG2D ligand expression and NK cell activity following CS exposure. These studies reveal potentially important roles for TLR receptors in development and progression of COPD by recognizing endogenous ligands and activating NK cells through NKG2D.

## Methods

### Mice

C57BL/6J mice (female, 8 to 10 wk old) were purchased from The Jackson Laboratory (Bar Harbor, ME). Mice with a single point mutation in the *Unc93b1* gene and are deficient in TLR3/7/9 signaling [C57BL/6-*Unc93b13d*/Mmcd (*Unc93b1*)] [16] were obtained from the Mutant Mouse Regional Resource Centers (MMRRC). NKG2D-deficient (Klrk1^−/−^) mice were generated as described [17]. All of the experimental protocols were reviewed and approved by the Institutional Animal Care and Use Committee at the University of Cincinnati Medical Center (Protocol 06-04-07-01).

### Cigarette smoke exposure

Mice were exposed to either filtered, room air (FA) or cigarette smoke (CS) generated from 3R4F Kentucky Reference Cigarettes (University of Kentucky, Lexington, KY). As previously described [18], CS exposures were carried out with a TE-10z smoking machine attached to an exposure chamber (Teague Enterprises, Woodland, CA). The concentration of the smoke/air mixture was maintained at 150 ± 15 mg/m^3^ total suspended particulates. Particulate concentrations were determined by weighing vacuum-drawn total particulate deposition onto filters connected to the chambers. Mice were exposed whole body in the exposure chamber for 4 h/d, 5 d/wk for 6 months.

### Nucleic acid isolation and quantification from bronchoalveolar lavage (BAL)

Mice were euthanized with an i.p. injection of sodium pentobarbital and the lungs were lavaged with two 1-ml aliquots of HBSS (minus Ca^2+^ and Mg^2+^, pH 7.2, 37°C; Invitrogen) containing 200U RNAse inhibitor (Qiagen). Samples were centrifuged at 300 x g for 10 mins to pellet cellular debris. Nucleic acids from the sample supernatants were analyzed for RNA and DNA using Qubit fluorometric quantitation (Invitrogen). Total nucleic acid concentrations were read on a Qubit 2.0 Fluorometer according to manufacturer’s protocols (Invitrogen).

### Quantification of nucleic acids following exposure of epithelial cells to cigarette smoke extract

Cigarette smoke extract (CSE) was generated by bubbling smoke from one standard reference cigarette into 10 ml PBS through a 50-ml fritted impinger at a flow rate of 350 ml/min. The resulting extract was passed through a 0.22-mm filter and was considered 100% CSE. The CSE was adjusted to pH 7.4 by the addition of NaOH. Mouse lung epithelial cells (MLE-15) (provided by Dr. Jeffrey Whitsett, Cincinnati Children’s Hospital [19]) were grown in MLE media (DME/F12 with 2.5 mM l-glutamine and 15 mM Hepes [HyClone, Waltham, MA] containing 2% FBS, 1 x Insulin-Transferrin-Selenium-X [Sigma], 10 nM β-estradiol [Sigma], 10 nM Hydrocortisone [Sigma]). Cells were added to a 48-well plate and allowed to grow until ~80% confluent. Media was replaced with MLE media containing CSE concentrations as described in the text and allowed to incubate for 16 hrs at 37°C and 5% CO_2_. Supernatant was collected following the incubation and total nucleic acid concentrations were calculated using Qubit fluorometric quantitation and read on a Qubit 2.0 fluorometer according to manufacturer’s protocols (Invitrogen).

### Leukocyte enumeration in BAL

Lungs were lavaged two times with 1 ml of 1× HBSS. The first bronchoalveolar lavage (BAL) return was centrifuged at 300 × *g* for 10 min, and the supernatant was removed and stored at −80°C until assayed. The remaining cell pellet was combined with the second BAL return and centrifuged at 300 × *g* for 10 min. The cell pellet was resuspended in 1 ml 1× HBSS containing 2% fetal bovine serum. Total cell counts were determined with a hemocytometer. Differential leukocyte counts (>300 cells) were determined on Hemacolor-stained (EM Science, Gibbstown, NJ) cytospin slides (Cytospin3; Shandon Scientific Ltd, Waltham, MA).

### Lung fixation, histology, MLI

For histology analysis, mouse lungs were fixed in buffered formalin as previously described [20]. The mean linear intercept (MLI), a measure of interalveolar distance, was determined as previously described [21]. Focal areas of pulmonary inflammation were classified as slight (<25 cells), mild (25–100 cells), moderate (7500–20,000 µm^2^), or severe (>20,000 µm^2^) and according to the morphological features associated with the inflammation. Inflammation scores for individual mice were obtained by summing the instances of inflammation weighted for severity as follows: slight, 1; mild, 2; moderate, 4; and severe, 8 as described by our lab [22].

### Stimulation of TLR reporter cell lines

Mouse HEK-Blue TLR3, TLR7, and TLR9 expressing HEK293 reporter cell lines were grown in DMEM supplemented with 10% FBS and selective antibiotics according to the manufacturer’s protocol (InvivoGen). MLE-15 cells, grown in MLE media, were added to a 6-well plate and allowed to grow until ~80% confluent. Once proper cell density was obtained, the media was removed and replaced with HEK-Blue media containing the concentration of cigarette smoke extract (CSE) indicated in the text. These cells were allowed to incubate overnight at 37°C and 5% CO_2_. The following day 180 µl of the supernatant was removed, added to the HEK-Blue reporter cells, and incubated overnight. In addition, some cells were pretreated with Benzonase (50 U/mL, 37°C, 30 min) endonuclease to degrade the nucleic acids in the media before TLR stimulation (Sigma). Secretion of alkaline phosphatase was analyzed using HEK-Blue detection medium according to the manufacturer’s protocol (InvivoGen).

### Natural Killer cell isolation and stimulation

Mice were euthanized with an i.p. injection of sodium pentobarbital followed by exsanguination. Lungs were perfused with 6 ml 1× PBS containing 0.6 mM EDTA. Lungs and spleens were withdrawn aseptically, and leukocytes were isolated as previously described [5]. Leukocytes were resuspended in cRPMI (RPMI 1640 with 2.05 mM l-glutamine [HyClone, Waltham, MA] containing 10% FBS, 1% sodium pyruvate, 100 U/ml penicillin, 100 µg/ml Streptomycin, and 1× nonessential amino acids [MP Biomedicals, Solon, OH]) followed by a 20-min plastic adherence plating step at 37°C and 5% CO2 that greatly reduces the presence of contaminating adherent cells. After plating, the remaining leukocytes were enriched for NK cells by positive selection, following the manufacturer’s protocol for the Dynabead FlowComp Mouse CD49b NK isolation kit (Invitrogen). After enrichment, NK cells were >60% pure. The remaining cells were stained with anti-NKp46 and sorted by flow cytometry for NK cells, resulting in a purity >99%. A total of 2.0 × 10^5^ cells in 100 µl cRPMI containing 20 U/ml mouse rIL-2 (PeproTech) was aliquoted per well into a 96-well round-bottom culture plate (Costar, Cambridge, MA) and cultured at 37°C and 5% CO2. Cells were rested 2 h then stimulated for 16 h with either R848 (5 µg/ml) (Invivogen), LPS (5 µg/ml) (Invivogen), IL-12 (10 ng/ml) (Peprotech), IL-18 (10 ng/ml) (R&D Biosciences), or IL-12 + IL-18 (10 ng/ml). Supernatants were harvested and assayed for IFN-γ production by ELISA (eBioscience).

### Flow cytometry for RAET1

In one set of studies, MLE-15 cells were treated with poly(I:C) (50 µg/ml), ODN1826 (1 µM), or PBS for 24hrs and cells were stained with anti-Raet1 antibody (clone 186107, R&D Systems) and analyzed by flow cytometry using a FACSCalibur (BD Biosciences, San Jose, CA). Live cells were selected by excluding 7-AAD positive cells (eBioscience). The data was analyzed using FlowJo software (Tree Star, Ashland, OR). To examine the contribution of individual TLR receptors in CSE-induced Raet1 induction, we exposed MLE-15 cells to 20% CSE, 20% CSE + TLR3 antagonist [(R)-2-(3-Chloro-6-fluorobenzo[b]thiophene-2-carboxamido)-3-phenylpropanoic acid] (Calbiochem) and the TLR9 antagonist ODN 2088 (Invivogen) or PBS for 24hrs and cells were stained with anti-Raet1 antibody and analyzed by flow cytometry as described above.

### RT-PCR

Frozen tissue was homogenized using a Tissumizer (Tekmar), and total RNA was isolated with TRIzol reagent (Invitrogen). DNase treatment to remove residual DNA was performed using the Turbo DNA-free kit (Ambion). Reverse transcription of total RNA was performed using the high-capacity cDNA Archive kit (Applied Biosystems). FAM-labeled probes used for RT-PCR were Raet1 (Mm04206137_gh), and Rpl32 (Mm02528467_g1) (Applied Biosystems). Quantitative RT-PCR was performed using TaqMan universal PCR master mix (Applied Biosystems) on an Applied Biosystems 7300 real-time PCR system. Expression of mRNA was quantified by the ΔΔ*C*_*T*_ method using *Rpl32* as the endogenous control.

### Statistics

Significant differences among groups were identified by t test or one-way ANOVA, wherever appropriate. Differences between means were considered significant at p < 0.05. Inflammation scores were transformed by taking the square root of the raw scores, and the total numbers of cells were transformed using Log_10_ of the raw counts. In every instance, the Student t test was used, normality was assessed using Kolmogorov-Smirnoff test, and homogeneity of variances was tested using SigmaPlot 10.0 to ensure that the assumptions of the Student t test were not violated. A p value of <0.05 was considered significant.

## Results

### NKG2D deficiency attenuates airspace inflammation and pulmonary remodeling associated with CS exposure

**Figure 1:**
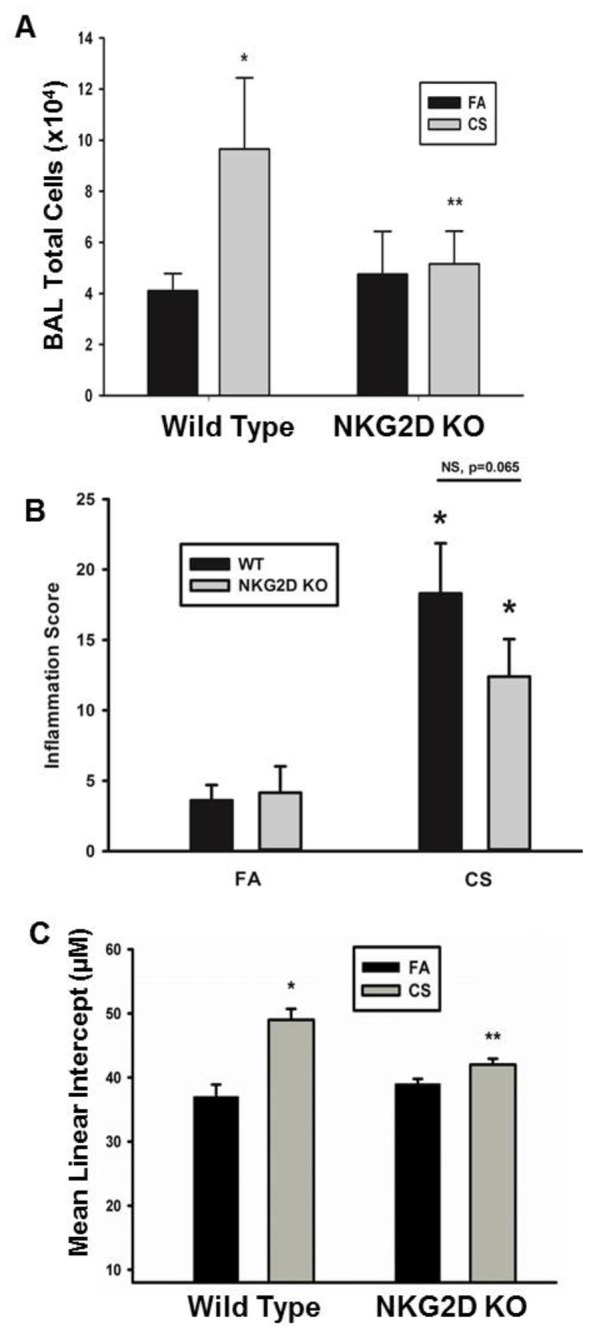
NKG2D is required for CS-induced pulmonary inflammation and alveolar destruction. WT and NKG2-deficient deficient mice were exposed to CS or FA for 6 months. (A) BAL was performed and cells were enumerated. > 95% of cells were macrophage and no differences observed in other cell types (data not shown). (B) Inflammatory aggregates surrounding the airways and vasculature were scored by number of occurrences and severity in H%E-stained lung sections. Inflammation scores were transformed by taking the square root of the raw scores, and the total numbers of cells were transformed using Log_10_ of the raw counts. * Significantly different than FA strain matched mice determined by Student t test. n = -6 mice. (C) Mean linear intercept was quantitated to define the average alveolar diameter. * Significantly different than FA strain matched mice determined by Student t test. ** Significantly different than CS-exposed wild type mice determined by Student t test. n = 5-6 mice.

Induction of NKG2D ligands on pulmonary epithelial cells is associated with CS exposure and loss of lung function in humans and mouse models. Additionally, ectopic, conditional expression of NKG2D ligands in pulmonary epithelial cells is sufficient to drive the development of airspace enlargement in transgenic mice [3]. We examined the effects of long-term CS exposure in NKG2D–deficient mice to further investigate the role of NKG2D in the increased inflammation and development of alveolar destruction. Figure **1A** shows that NKG2D deficiency results in a significant decrease in the number of cells recovered in the bronchoalveolar lavage (BAL) of CS-exposed mice compared to filtered air (FA)-exposed mice. The decreased inflammatory response was non-specific as both macrophage and neutrophil accumulation were decreased by similar ratios (data not shown). In contrast to BAL cellularity, histological examination revealed that CS exposure elicited peribronchial and perivascular inflammation in both wild-type mice and NKG2D-deficient mice. Although there was a trend towards fewer inflammatory aggregates in the NKG2D-deficient mice compared to wild-type, the difference was not significant (Figure **1B**). Corresponding with the inhibition of inflammatory leukocytes in the airways and airspaces, NKG2D-deficient mice failed to develop airspace enlargement equivalent to that measured in wild-type mice following long-term CS exposure (Figure **1C**). Together, these data indicate that NKG2D is an important upstream regulator of CS-induced airspace inflammation and alveolar destruction.

### CS exposure elaborates nucleic acids and stimulates TLR signaling

The signaling pathways that elicit NKG2D ligand expression following CS exposure are unknown. Based on findings of Hamerman et al. demonstrating that stimulation of peritoneal macrophages with TLR agonists upregulates *Raet1* expression [23], we speculated that nucleic acid-binding TLRs (i.e., TLR3, TLR7/8, and TLR9) in/on epithelial cells may represent important sensors of pulmonary injury following CS exposure based on their ability to recognize exogenous mammalian RNA (TLR3 and TLR7) [24,25]and DNA (TLR9) [26].

**Figure 2:**
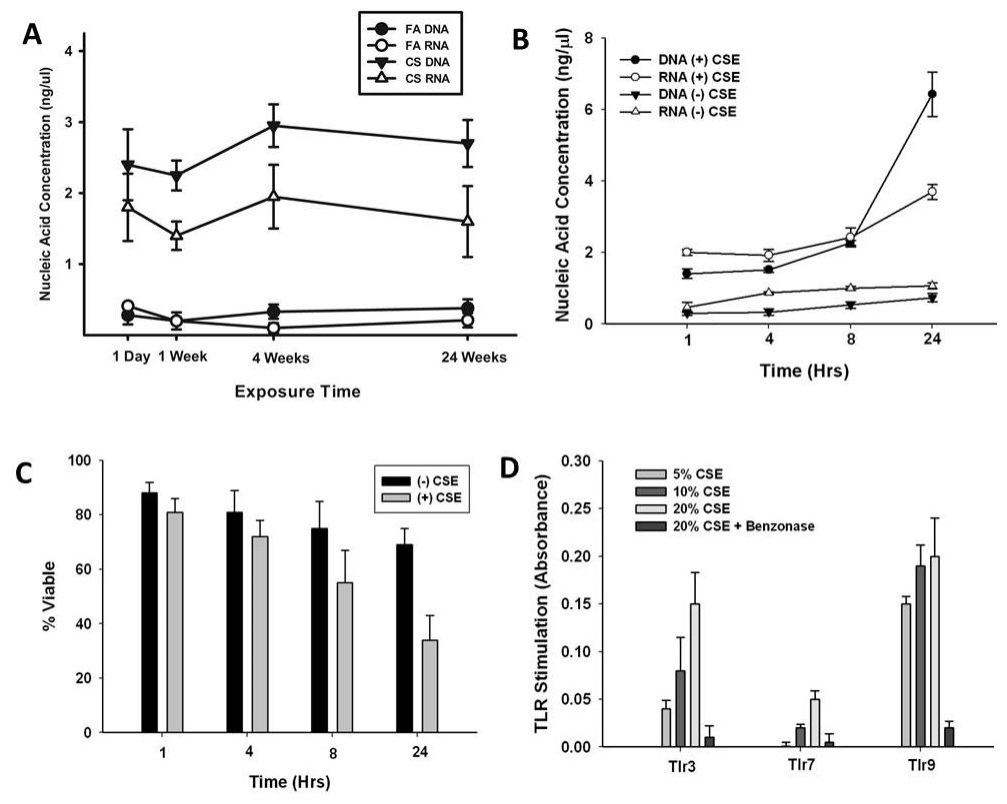
CS exposure induces nucleic acid release into the lung. (A) C57BL/6 mice were exposed to FA or CS and total nucleic acid in the BAL fluid was quantified using highly sensitive and highly specific Qubit fluorescent dyes. RNA and DNA levels were significantly greater in CS-exposed mice compared to FA-exposed mice at all times. n = 6 mice. (B) MLE-15 cells were exposed to 20% cigarette smoke extract (CSE) for up to 24 hours. RNA and DNA levels were quantified using highly sensitive and highly specific Qubit fluorescent dyes. RNA and DNA levels were significantly greater in CSE-exposed cells compared to PBS-exposed cells at all times as determined by Student t test. Data are means ± SEM of 3 independent experiments. (C) MLE-15 cells were exposed to 20% cigarette smoke extract (CSE) for up to 24 hours and cell viability was assessed using the MTT assay. Data are means ± SEM of 3 independent experiments. (D) MLE-15 cells were exposed to increasing concentrations of CSE for 24 hrs. Cell-free supernatant, with and without endonuclease (Benzonase) treatment, from exposed cells was applied to reporter cell lines expressing individual TLRs. TLR activation, as measured byNF-κB-induced SEAP activity, was assessed using QUANTI-Blue and by reading the absorbance at 620 nm. Data are shown as the mean ± SEM of three independent experiments.

First, we examined whether repeated CS exposure results in the elaboration of endogenous nucleic acids. Mice were exposed to FA or CS for up to six months and nucleic acids concentrations in the bronchoalveolar lavage (BAL) were quantitated. We found that mice exposed to CS had greater amounts of both RNA and DNA recovered from the BAL fluid than FA-exposed mice (Figure **2A**). The presence of RNA and DNA in the BAL was apparent as following an initial exposure and persisted for the entire six month exposure period. To further clarify the role of CS on the release of cellular nucleic acids, we exposed a mouse lung epithelial cell line (MLE-15) to cigarette smoke extract (CSE) and observed time-dependent increases in cell-free free nucleic acids (Figure **2B**). The release of nucleic acids was correlated with increased cell death suggesting that dead and dying cells are the source of the nucleic acids (Figure **2C**). Next, we examined the ability of cell culture media from CSE-exposed MLE-15 cells to stimulate TLR3, 7, and 9 HEK-Blue TLR specific transfected cell lines. Figure **2D** demonstrates a dose-dependent increase in the activation of all three reporter cell lines with TLR9 activation>TLR3>TLR7. To test whether these responses were attributed to nucleic acid –mediated activation, we treated CSE media with a potent endonuclease prior to incubation with the reporter cell lines. This experiment clearly demonstrates that the effects on TLR stimulation are mediated through nucleic acids.

### TLR3 and TLR9 agonists induce NKG2D ligand expression in pulmonary epithelial cells

**Figure 3:**
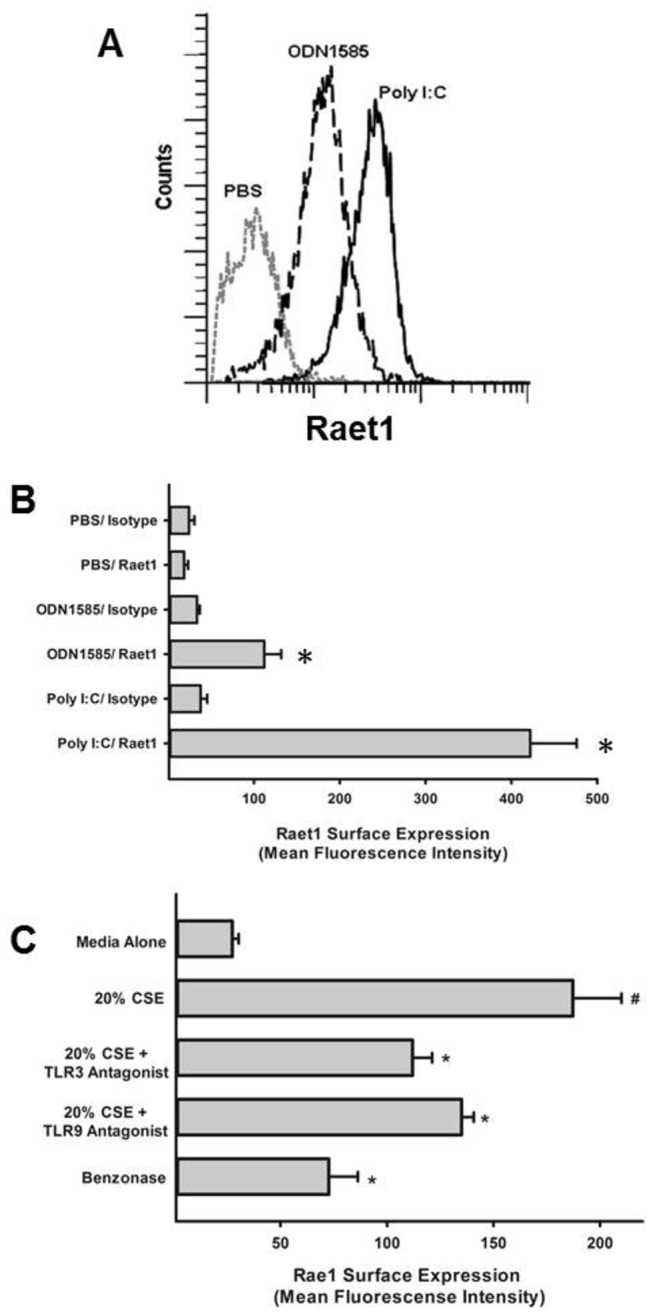
Induction of NKG2D ligands by TLR ligands on mouse pulmonary epithelial cells. (A) MLE-15 cells were treated with synthetic RNA (50 µg/ml poly(I:C)), DNA CpG (1 µM ODN1826) or PBS for 24 h. Cell surface Raet1 expression was assessed by flow cytometry. Histograms are representative of three independent experiments. (B) Quantification of Raet1 induction on MLE-15 cells. Data are shown as the mean ± SEM of three independent experiments. * Significantly different than PBS-treated cells as determined by Student t test. (C) CSE-induces Raet1 expression on MLE-15 cells which can be inhibited with TLR3 antagonists [(R)-2-(3-Chloro-6-fluorobenzo[b]thiophene-2-carboxamido)-3-phenylpropanoic acid] and the TLR9 antagonist (50 nM of ODN 2088), and degradation of nucleic acids using an endonuclease (50 U/mL Benzonase). Data are shown as the mean ± SEM of three independent experiments. # Significantly different than PBS-treated cells as determined by Student t test. * Significantly different than 20% CSE-treated cells as determined by Student t test.

Given our preliminary data that NKG2D is required for CS-induced COPD pathologies in mice (Figure **1**) and that NKG2D ligand expression in macrophages is induced by TLR agonists [23], we examined whether TLR stimulation upregulates NKG2D ligand expression on pulmonary epithelial cells. We exposed MLE-15 cells to TLR3, TLR7, and TLR9 ligands and assessed cell surface expression of the NKG2D ligand Raet1. Poly(I:C) (TLR3) and ODN1826 (TLR9) induced RAET1 expression on the surface of MLE-15 cells (Figure **3A and 3B**). No response was detected following stimulation with the TLR7/8 ligand ssRNA40 (not shown). We next examined the ability of media from CSE-exposed epithelial cells to induce cell surface NKG2D ligand expression. In addition, we examined the relative contribution of TLR3 and TLR9 to NKG2D ligand upregulation. Figure **3C** demonstrates that CSE is sufficient to induce NKG2D ligand expression and that the TLR3/dsRNA complex inhibitor [(R)-2-(3-Chloro-6-fluorobenzo[b]thiophene-2-carboxamido)-3-phenylpropanoic acid] and the TLR9 antagonist (ODN 2088) both inhibit CSE-induced ligand expression. Finally, to confirm that ligand expression was attributed to nucleic acid –mediated activation, we treated CSE media with a potent endonuclease and demonstrate that effects of CSE are mediated through nucleic acids. Together, these findings suggest nucleic acids liberated from CS-exposed cells can have a direct effect on pulmonary epithelial cells to express activating signals for presentation to natural killer cells.

### TLR3/7/9 signaling mediates NK cell function, NKG2D ligand expression and airspace enlargement in a mouse model of COPD

**Figure 4:**
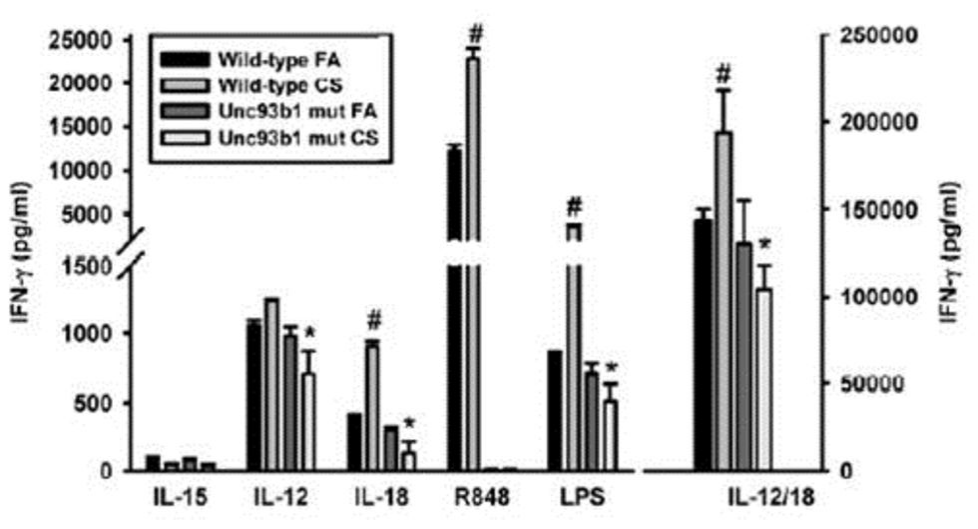
TLR3/7/9 mediates CS-induced NK cell hyperresponsiveness. Lung leukocytes were isolated from FA- and CS-exposed mice, NK cells were purified, and stimulated with 10 ng/ml IL-12 or IL-18 alone, or in combination for 20 h. NK cells were also cultured with TLR7/8 agonist R848 (100ng/ml) as a negative control for TLR7 mutation and LPS (10ng/ml) for a positive control for TLR4 responsiveness. IFN- γ was measured by ELISA. Values are presented as means ± SEM. # Denotes values significantly different from wild-type FA. *Denotes values that are significantly different from wild-type CS exposure determined by Student t test. *p* < 0.05. Data are representative of three independent experiments. n= 4 mice per group.

Chronic cigarette smoke exposure “primes” NK cells to produce more IFN-γ and exhibit enhanced cytotoxicity after stimulation [20]. More recent studies have demonstrated that NKG2D is required for these enhanced NK cell effector functions [5]. The mechanisms that lead to the expression of these ligands are unknown. In this study we have shown that TLR stimulation enhances expression of RAET1 on the surface of mouse lung epithelial cells (Figure **3**). To determine the extent that endogenous TLR ligands contribute to CS-induced pathologies, we utilized a unique C57BL6 mutant mouse that has a mutation in the *Unc93b1* gene that is crucial for TLR3, 7, and 9 signaling functions [16]. We exposed C57BL6 or *Unc93b1* mutants to FA or CS and examined NK cell responses to cytokines and TLR agonists. Figure **4** demonstrates that disruption of TLR3/7/9 signaling eliminates the development of NK cell hyperresponsiveness to cytokines and LPS, a TLR4 ligand. We also exposed the NK cells to a TLR7/8 agonist (R848) to verify that this TLR pathway was eliminated in the *Unc93b1* mutant mice.

**Figure 5:**
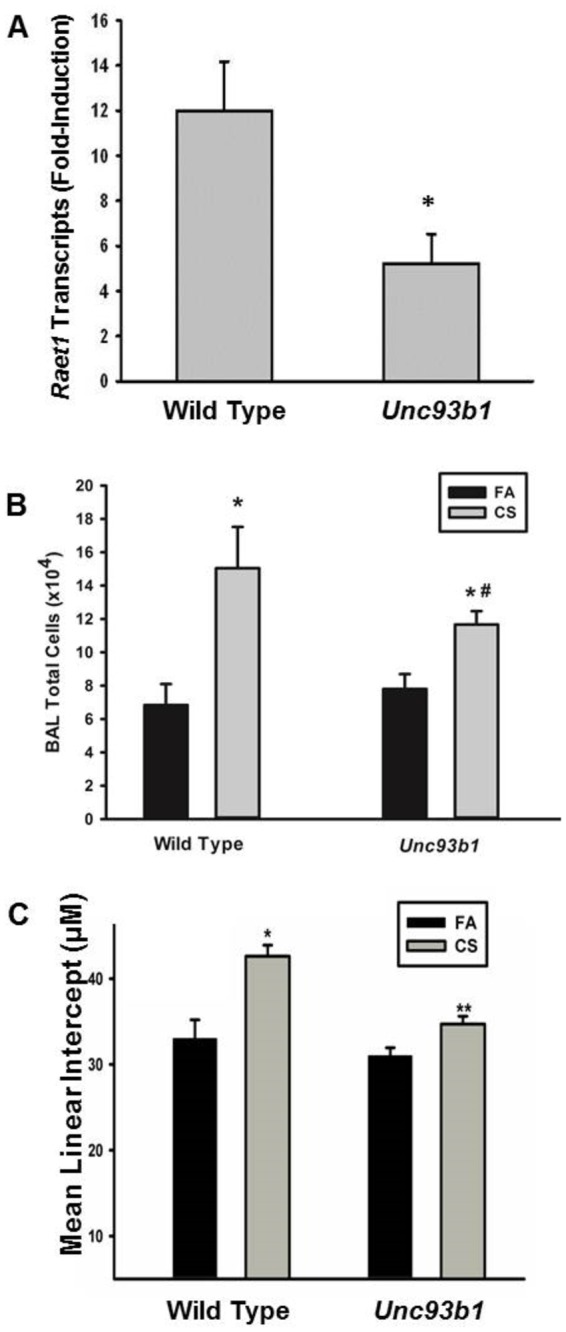
TLR3/7/9 signaling mediates alveolar destruction, pulmonary inflammation, and maximal NKG2D ligand induction in CS-exposed mice. *Unc93b1* mutants (TLR3/7/9 signaling deficient) and WT C57BL/6 mice were exposed to CS or FA for 6 months. (A) *Raet1*expression was measured by RT-PCR from the lungs of exposed mice. *Denotes value significantly different from wild-type CS exposure determined by Student t test. p < 0.05. n = 4-6 mice. (B) BAL was performed and cells were enumerated. > 95% of cells were macrophage and no differences observed in other cell types (data not shown). * Significantly different than FA strain matched mice determined by Student t test. ^#^ Significantly different than CS-exposed wild type mice determined by Student t test. (C) Airspace enlargement was assessed by Mean linear intercept. * Significantly different than FA strain matched mice determined by Student t test. ** Significantly different than CS-exposed wild type mice determined by Student t test. *p* < 0.05. n = 4-6 mice.

Next, we sought to determine whether TLR recognition and signaling was an integral component of NKG2D ligand induction in the lung following long-term CS exposure. We exposed C57BL6 or *Unc93b1* mutants to FA or CS for 6 months and measured *Raet1a-e* transcript expression in the lung. Wild type mice exposed to CS have a significant increase in the expression of *Raet1* transcript. In contrast, mice that lack functional TLR3/7/9 fail to express higher levels of *Raet1* following CS (Figure **5A**). There were no significant differences in baseline expression between the BL6 and the *Unc93b1* mutant. To further assess the role of these TLRs in the progression of COPD-related pathologies we measured pulmonary inflammation and the development of airspace enlargement of CS-exposed WT and *Unc93b1* mutants. These studies show that Unc93b1 mice develop increased accumulation of BAL cells in response to CS exposure but also display a significant attenuation of BAL inflammation compared to CS-exposed wild-type mice (Figure **5B**). In agreement with these findings, we found that WT mice exhibited increased airspace enlargement following CS exposure and this effect was attenuated in the *Unc93b1* mutant mice (Figure **5C**). These findings suggest that TLR activation is as significant a contributor as NKG2D to COPD pathologies and may be a potential upstream mediator of the effect.

## Discussion

The lung is a dynamic organ that constantly counteracts the harmful effects of exposures to noxious particles and airborne pathogens. The airway epithelia are located at the interface between airway exposures and the innate immune system. Airway epithelial cells are adept at sensing and responding to foreign pathogens through a diverse array of pattern recognition receptors known as Toll-like Receptors (TLRs) [27]. TLR activation releases inflammatory, chemotactic, and antimicrobial mediators which are important in combating the presence of foreign pathogens. The role of TLRs in the response of the airway epithelium to chronic cell injury remains to be elucidated. The same TLR receptors that identify pathogen-associated molecular patterns (PAMPs) can be activated by endogenous molecules that are released in response to cell injury. These endogenous molecules are known as damage-associated molecular patterns (DAMPs). While cigarette smoke (CS) has been shown to cause necrotic death in a range of epithelial cell lines [28,29] there are no reports describing CS-induced release of DAMPs that activate and signal through endosomal TLRs (i.e., TLR3, TLR7/8, and TLR9). Based on these findings, we hypothesized that repeated stimulation of nucleic acid-sensing TLRs is a potentially important pathway involved in NK cell activation associated with CS exposure and COPD pathologies. In the current study, we found that nucleic acid levels found in the BAL of CS-exposed mice were elevated compared to FA-exposed mice. More specifically, we found that CS exposure causes the release of TLR3, 7, 9 stimulating molecules utilizing sensitive TLR-reporter cell lines. Using mice that are deficient in TLR3/7/9 signaling, we show that TLR3/7/9 stimulation plays a significant role in the inflammation and airspace enlargement associated with CS-exposure.

The development of COPD pathologies involves a complex coordination between cellular subsets and released cellular mediators. The use of genetically modified mice provides a powerful tool for elucidating the contributions of NK cell mediators to disease progression. Kang et al. reported that CS-exposed mice deficient in IL-18, a strong NK cell stimulatory cytokine, had attenuated COPD pathologies following influenza infection [30]. In addition, lung-specific induction of IFN-γ is sufficient to induce COPD symptoms including, pulmonary emphysema and inflammation [31]. In this study, mice deficient in NKG2D, a cytotoxic lymphocyte activating receptor, showed attenuated CS-induced inflammation in the airspaces, around the airways and blood vessels, and were protected from extensive alveolar remodeling. These results support previous findings that conditional expression of RAET1 in pulmonary epithelial cells is sufficient to cause severe pulmonary emphysema [3]. Interestingly, no evidence of pulmonary inflammation was associated with alveolar destruction in the RAET1 inducible model of airspace enlargement. The lack of inflammation in the RAET1 transgenic indicates that additional mediators are involved in the CS- induced NKG2D-mediated cell-mediated pulmonary remodeling.

It is well established that CS exposures initiate necrotic and apoptotic cell death in vivo [32]. In the current study, we show that after CS-exposure TLR ligands are released, a likely indication that cells are undergoing some level of necrotic cell death most likely originating from epithelial cell, macrophage and neutrophils. As TLR ligands are potent pro-inflammatory signals, they represent likely mediators of the enhanced inflammation during CS-exposure. Furthermore, NK cells from CS-exposed mice deficient in TLR3/7/9 signaling lack the enhanced response to stimulation seen in CS-exposed WT mice. Activation of NK cells by TLR ligands occurs indirectly through accessory cells, therefore, the effects of TLR activation on COPD disease progression is likely upstream of NKG2D engagement.

In the context of normal tissue surveillance, NKG2D ligand expression indicates cellular stress and represents a self-identification mark for cytotoxic lymphocytes. DAMPs also represent “danger signals” and it is not surprising that we were able to link the activation of TLRs and the expression of NKG2D ligands in the context of chronic pulmonary injury in COPD. The aberrant expression of NKG2D ligands has been linked with a number of autoimmune diseases which in turn have been linked to a heightened TLR activation, including rheumatoid arthritis [33,34], coeliac disease [35]and autoimmune diabetes [36]. Zhou et al. have linked TLR3 activation and NKG2D activation to epithelial cell homeostasis in the small intestine [37]. This study demonstrated that treatment with high doses of polyI:C (a TLR3 ligand) induced small intestinal injury via the upregulation of NKG2D ligands and cell-mediated cytotoxicity directed at the intestinal epithelium. While the study by Zhou et al. examines the same pathways presented here, there are several important distinctions in the mechanisms that mediate the respective pathologies. The intestinal epithelial cell breakdown by polyI:C treatment is an acute model that causes massive cell death mediated by CD8aa lymphocytes which acquire NKG2D signaling via IL-15 dependent mechanisms. In contrast, our model involves chronic, low-level injury in response to a common agonist (CS), the persistent release of small amounts of endogenous nucleic acids, NK cell phenotypic changes and the gradual destruction of the lung architecture. However, both of these studies reveal that aberrant expression of ligands for activating NK cell receptors can be mediated by TLR receptors on epithelial cells and may contribute to form of breakdown in immune tolerance by eliciting exaggerated responses to endogenous cellular products.

In patients with rheumatoid arthritis, the expression of TLR2, TLR3 and TLR7 is significantly enhanced in synovial fibroblasts [38]. Additionally, necrotic cells from synovial fibroblasts of rheumatoid arthritis patients stimulate the release of cytokines through TLR3 activation [39]. In a mouse model of autoimmune diabetes, mice that are deficient in TLR 9 but not TLR 3 are 50% less likely to develop pathology [40]. Based on the data presented in this study, it seems likely that the progression of COPD is a result of a coordinated effort between TLR activation and the enhanced expression of NKG2D ligands.

**Figure 6:**
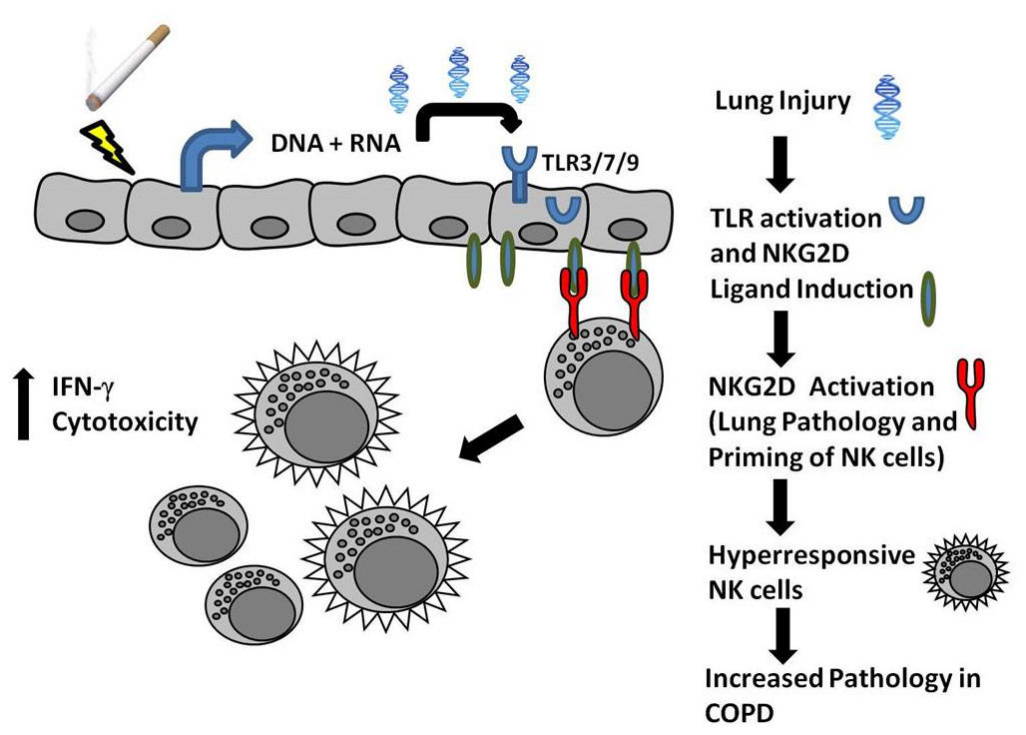
Proposed mechanism of CS-induced expression of NKG2D and its role in COPD CS causes localized damage of lung epithelial cells leading to the release of endogenous TLR ligands. These ligands are sensed by TLR receptors on neighboring epithelial cells resulting in the induction of NKG2D ligands. Persistent expression and engagement of NKG2D ligands causes NK cells to be hyperresponsive to future activation. Important to the pathogenesis of COPD, enhanced NK cell activation may lead to increased macrophage or monocyte and neutrophil recruitment and activation, destruction of lung tissue and alveolar apoptosis.

Together, our data identifies key components in the etiology of COPD. Figure **6** represents a potential model for the role of NK cells in the development of COPD pathologies. Briefly, CS-exposure damages pulmonary epithelial cells causing the release of endogenous TLR ligands. The effect of localized CS-induced cell damage is magnified by the increase in pulmonary inflammation and induction of NKG2D ligands on surrounding cells following TLR engagement. The expression of NKG2D ligands leads to the targeted destruction of nearby pulmonary tissue exacerbating the initial exposure. Similar to chronic inflammatory diseases, smoking is a persistent habit resulting in the continued release inflammatory and cytotoxic mediators. The repeated expression of NKG2D ligands and release of stimulatory cytokines results in NK cells that are hyperresponsive to future stimulation. Ultimately, persistent pulmonary injury becomes a driving force of the imbalance between injury and repair that occurs subtly over a long period of smoke exposure.
